# Profile of Volatile Aroma-Active Compounds of Cactus Seed Oil (*Opuntia ficus-indica*) from Different Locations in Morocco and Their Fate during Seed Roasting

**DOI:** 10.3390/foods9091280

**Published:** 2020-09-11

**Authors:** Issmail Nounah, Malika Chbani, Bertrand Matthäus, Zoubida Charrouf, Ahmed Hajib, Ina Willenberg

**Affiliations:** 1 Laboratory of Plant Chemistry and Organic and Bio-Organic Synthesis, Faculty of Sciences, Mohammed V University of Rabat, Rabat 10000, Morocco; nounahissmail@gmail.com (I.N.); malikachbani96@gmail.com (M.C.); zcharrouf@yahoo.fr (Z.C.); hajib.ahmed1@gmail.com (A.H.); 2 Working Group for Lipid Research, Department of Safety and Quality of Cereals, Max Rubner-Institut (MRI), 32756 Detmold, Germany; bertrand.matthaeus@mri.bund.de

**Keywords:** headspace GC-MS, olfactometry, *Opuntia ficus-indica*, roasting, volatile compounds

## Abstract

Volatile compounds from oils extracted from cactus seeds (*Opuntia ficus-indica*) of five regions of Morocco were analyzed by dynamic headspace-GC/MS. Aroma active compounds were characterized by olfactometry. A total of 18 compounds was detected with hexanal, 2-methyl propanal, acetaldehyde, acetic acid, acetoin and 2,3-butanedione as most abundant. Olfactometric analysis showed that those compounds are aroma active; therefore, cactus seed oil flavor can be attributed to those compounds. Moreover, the effect of roasting of cactus seeds on the composition of volatile compounds in the oil was investigated. Especially the concentration of compounds known as products from the Maillard reaction increased significantly with roasting time such as furfural, furan, 3-methyl furan, 2-butanone, thiophene, 2, 3- dithiabutane, methyl pyrazine, 2-methyl pyrimidine, 2-metoxy phenol, dimethyl trisulfide and 5-methyl furfural.

## 1. Introduction

*Opuntia ficus-indica* originally came from the tropical and subtropical regions of Central and South America and nowadays the plant is located in many arid and semi-arid areas of the world with occurrences in Spain, the Mediterranean Basin, Africa, the Middle East and Asia. In North Africa, it was introduced in the 16th century. The plant belongs to the family Cactaceae, and as a robust plant, it can be grown well in hot and dry areas. *O. ficus-indica* is interesting at an environmental level because the cultivation allows protection of the grounds against erosion, fighting against desertification, slowing down the rate of degradation of deforested soils and conserving biodiversity. In view of the environmental changes under way, the cactus pear could be considered an option as a carbon sink, absorbing and holding excess carbon dioxide in areas where the plant can be established but where nothing else will grow [1].

Today, *O. ficus-indica* is cultivated on an area of about hundred-fifty thousand hectares in Morocco [2], with an annual production of fruits up to 20 tons dry matter/hectare/per year under optimal conditions [2] or a mean production of 8 tons of fruits per hectare [3]. The cultivation of prickly pear is low in investment, and it can generate an important income by using the different parts of the plant for the preparation of food and human nutrition. These products will play an important socio-economic role for farmers and rural populations and will contribute to sustainable development in rural areas [3,4].

The edible part of the fruit contains up to 300 small seeds [5], which amount can vary from 30% to 40% on a dry weight basis [6], with a weight between 15 and 20 mg [7]. Some years ago, these seeds were usually discarded even though proper utilization of these byproducts could lead to an important new source of oil and meal [8]. Although the oil content of the seeds is comparably low with amounts between 5 and 15%, the resulting oil has a valuable fatty acid composition rich in unsaturated linoleic acid (50–65%) and oleic acid (15–24%) [6,9,10,11]. The oil is mainly used in cosmetics due to the high price resulting from the time-consuming and laborious process of production of the small kernels. However, the oil is also suitable for human consumption due to the fatty acid composition similar to sunflower or grapeseed oil and γ-tocopherol as main antioxidant compound [12].

Virgin cactus seed oil is produced in small cooperatives in Morocco by use of a screw press to extract the oil from the seeds and filtration or sedimentation to purify the oil from solid plant material. The oil is characterized by a flavor resembling that of strawberries, watermelons, honeydew melons, fig, banana, or citrus [13], but nothing is known about the composition of the volatile and aroma active compounds that are responsible for these perceptions.

The interest in the pattern of volatile compounds of vegetable oils has increased significantly in recent years because volatile compounds directly affect the sensory quality of edible oils. In addition, the knowledge about the pattern of volatile compounds can help to support the sensory assessment of edible oils by analytical tools [14,15]. Several characteristic sensory perceptions can be explained by the occurrence of volatile compounds such as musty or fusty by the presence of 2-methylpropanol as well as 2- and 3-methylbutanal as microbial degradation products [16] or hexanal, octanal, and heptanal as indicators for oxidative degradation [17]. Further on, the pattern of volatile compounds can give important information about the processing of the oils [18,19,20,21].

Several investigations regarding the volatile compounds of fruits from *O. ficus-indica* have been published. Di Cesare et al. [22] and Di Cesare and Nani [23] showed that the composition of the volatile compounds of fruits depends on the variety, climatic conditions, and the method utilized for juice extraction. Arena et al. [24] identified sixteen volatile compounds extracted from the fruits by SMPE with (E)-2-hexen-1-ol and hexan-1-ol amounted to about 80% of the total weight of the extracts and (E,Z)-2,6-nonadien-1-ol and 2-methyl-butanoic acid methyl ester as most aroma active compounds. The strong effect of storage on the profile of volatile compounds extracted by SPME from pealed prickly pears was shown by Agozzino et al. [25] who mainly found an increase of two alcohols (2-nonen-1-ol and 2,6-nonadien-1-ol) and the corresponding aldehydric derivatives as reaction products from oxidation and hydrolysis. Rodrígues et al. [26] investigated the volatile compounds of the fruit pulp of different Opuntia species and showed undecane, phenylethyl alcohol, and pentyl acetate as major volatile constituents of *O. ficus-indica*. Very recently Andreu-Coll et al. [27] extracted a total of 35 compounds from *O. ficus-indica* fruits: 10 aldehydes (for example, 2,6-nonadienal), 8 terpenes (*β*-myrcene), 7 esters (methyl-3-hexenoate), 6 alcohols (nonanol), 1 ketone (1-penten-3-one), 1 linear hydrocarbon (5-undecene), and 1 terpene (linalool). In fresh and dried fruits of *Opuntia dilleniid,* 56 and 43 volatile compounds, respectively, were identified with esters (6.38%), aldehydes (41.79%), alcohols (17.82%), ketones (13.39%), and alkanes (9.20%) as main classes of compounds. Hexanal, octanal, octanol, nonanal, (E)-2-decenal, and hexadecane were present at the highest concentrations [28].

Although cactus seed oil is an increasingly interesting product, there has not yet been any research been published that describes the profile of the volatile compounds. However, it would be important to know more about the composition of the volatile compounds from cactus seed oil. Today, oil seeds are sometimes roasted before oil processing to give the resulting oil a pleasant taste and smell. The roasting process can change the composition of the volatile compounds by the formation of Maillard reaction products from the reaction of reducing sugars and amino acids. Information about the influence of roasting on the profile of volatile compounds would be useful for supporting the sensory evaluation of the oil.

Therefore, the aim of the present work was to characterize for the first time the profile of volatile aroma active compounds from cold pressed cactus seed oil extracted from seeds obtained from five different geographical origins, and different times of roasting at 110 °C, respectively. In addition, the presence of volatile compounds of the oil was compared to compounds extracted from the crushed seeds and the press cake obtained as residue from oil processing to verify whether the compounds in the oil were formed after the pressing.

## 2. Materials and Methods

### 2.1. Samples

**Figure 1:**
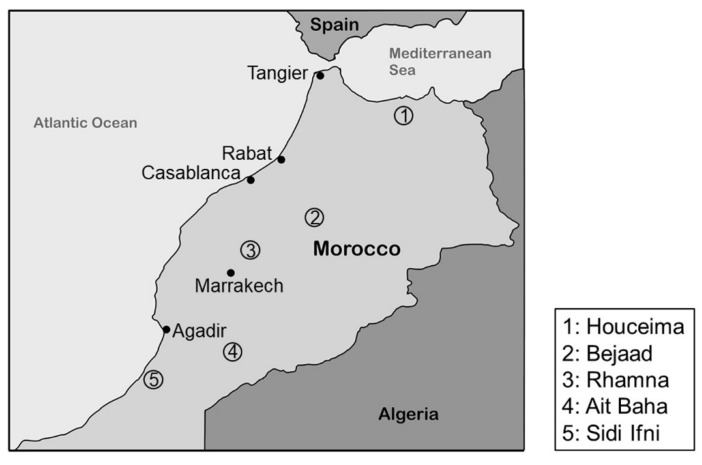
Location of the evaluated cactus seed sites of production in Morocco.

Cactus fruits used for this investigation were harvested between June and August 2017 at different locations in Morocco: 2 samples from Bejaad (two farms) (32°46′15″ N, 6°23′28″ W; elevation: 680 m), 2 samples from Ait Baha (two farms) (30°4′7.9″ N, 9°9′10″ W; elevation: 604 m), 1 sample from Rhamna (one farm) (32°28′12″ N, 7°57′29″ W; elevation: 491 m), 1 sample from Houciema (one farm) (35°14′41″ N, 3°55′60″ W, elevation: 133 m), and 1 sample from Sidi Ifni (one farm) (29°22′45″ N, 10°10′17.6″ W; elevation: 0 m) (Figure 1). In some cases, the seeds were prepared from two farms. From each farm the same plant (Species: *Opuntia ficus-indica*) and cultivar was used for the preparation of the seeds from the fruits. All samples studied were from the thorny variety.

### 2.2. Roasting of Cactus Seeds

For the study of the effect of roasting on content and composition of volatile compounds, only seeds from Sidi Ifni were used. Roasting was carried out at 110 ± 5 °C for 10, 20, 30 or 40 min using a roaster with continuous mixing of the material. The temperature was monitored using a Testo 945 thermometer sensor (Testo, Casablanca, Morocco).

### 2.3. Extraction of Cactus Seed Oil by a Screw Press

The extraction of the oil from the seeds was carried out using a CA59 G screw press (IBG Monforts GmbH & Co., Mönchengladbach, Germany). After extraction, the oil was left in the fridge over night to allow plant particles to settle. Afterwards, the oil was centrifuged for 15 min at 3000 rpm, and the oil was separated from the sediment. The purified oil was stored in the dark at 4 °C until analysis.

### 2.4. Reagents

The following compounds were used as analytical standards for the identification and quantification of distinct compounds in cold pressed cactus seed oil samples: acetoin, 3-methyl butanal, hexanal, 2-methyl propanal, pentanal, 2-methyl butanal, 3-methylbutan-1-ol, 2,3-butanedione and 1-hexanol (Sigma-Aldrich, St. Louis, MO, USA), acetic acid (Carl Roth, Karlsruhe, Germany), ethyl acetate, isopropyl alcohol, ethanol, and acetone (Merck, Darmstadt, Germany). Stock solutions (1000 mg/kg) and standard solutions (1 mg/kg, 10 mg/kg) were prepared in refined rapeseed oil. A mix of heptane, nonane, decane, and undecane (each 1 mg/kg; Sigma Aldrich (St. Louis, MO, USO) in refined rapeseed oil was used as internal standard for the GC-MS experiments. Refined oil was used as fresh oils do not contain high amounts of volatile compounds. Moreover, the refined rapeseed oil used was analyzed before preparing the standard solutions in order to prove that no volatiles from the refined rapeseed oil are interfering with the analysis of the volatile compounds from cactus seed oil.

### 2.5. Dynamic Headspace GC-FID

The extraction of the volatile compounds from cactus seed oil was done with a PTA 3000 dynamic headspace system using 200 mg of oil weighed into a 20 mL headspace vial. The volatile compounds were purged from the oil by a stream of nitrogen (0.7 bar, 20 mL/min) for 20 min at 80 °C. Carbon dioxide was used as cooling device for trapping the purged volatile compounds at an on-line Tenax trap. By heating the trap to 200 °C for 10 min, the accumulated volatiles were transferred via a heated (200 °C) uncoated fused silica transfer line to the GC system. For the separation of the volatile compounds a 5890 series II GC (HP Hewlett 5890 Packard Series II) was used equipped with a CPSil 19 fused silica capillary column (14% cyanopropyl-phenyl + 86% dimethylpolysiloxane), 60 m, 0.32 mm ID, 1 μm film thickness. The oven temperature was held for 5 min at 40 °C, then heated at 3 °C/min to 245 °C, and finally held isotherm for 10 min. By using a higher temperature for the oven than for the transfer line, it was ensured that the compounds come off the column faster and that no compounds remain on the column. Detection of the separated volatile compounds was carried out by a flame ionization detector operated at 280 °C with air, H_2_ and N_2_ as auxiliary gas. Gas flows were manually adjusted to ensure a continuous flame. A blank run, only consisting of air, was carried out before each new sequence. The evaluation and integration of the signals was performed by using Chemstation software. Identification of the components was done by comparison of retention time with that of the analytical standards.

For the quantitative analysis, the samples were analyzed in triplicate. Generally, the limit of detection is below 0.1 mg/kg. Quantification was carried out by a one-point calibration of 2-methyl propanal, 3-methyl butanal, hexanal, ethanol, pentanal, 2-methyl butanal, isopropyl alcohol, 3-methylbutan-1-ol, acetone, 2,3-butanedione, acetoin, acetic acid, ethyl acetate, 1-hexanol in a concentration of 10 mg/kg. Quantification of propane, acetaldehyde, pentane, and methyl isocyanide was done by use of ethanol standard.

### 2.6. Dynamic Headspace GC-MS of Volatile Compounds

According to method DGF-C-VI 20 [29], approximately 200 mg of cactus seed oil were exactly weighed into a 20 mL headspace vial. As internal standard two drops (approx. 40 mg) of a mixture of heptane, nonane, decane, and undecane (each 1 mg/kg) was added. The exact weight was recorded. As in the case of the dynamic headspace GC-FID, a PTA 3000 dynamic headspace system was used for collecting the volatile compounds with the same device settings (purge time: 20 min; temperature: 80 °C; purge gas: helium: pressure 0.7; purge flow: 20 mL/min; trap: on-line Tenax trap; trap temperature: −35 °C; cooling device: Peltier element with carbon dioxide as cooling material; transfer line from trap to GC: uncoated fused silica transfer line (200 °C)). The volatile compounds were released from the trap by heating the trap to 200 °C for 10 min, and the separation was achieved on a gas chromatographic system with a Trace 1300 Series GC (Thermo Scientific, Darmstadt, Germany) equipped with a CPSil 19 fused silica capillary column (14% cyanopropyl-phenyl + 86% dimethylpolysiloxane), 60 m, 0.32 mm ID, 1 μm film thickness. For the separation of the compounds, the same temperature program as for the GC-FID experiments was used. An ISQ Mass spectrometer (Thermo Scientific, Darmstadt, Germany) connected to the GC by a transfer line, tempered at 200 °C, was used for the detection of the compounds. A second port was connected to an olfactometry detection port. The detection of the compounds was achieved by electron ionization (EI) at 230 °C in positive mode. The scan of the ions from 35 to 300 m/z was done by the instrument in a time of 0.2 s. Each new sequence was started with a blank run, only consisting of air. The analysis of the HS-GC-MS data was performed by using Xcalibur 2.2. Identification of the components was done by comparison of retention time and MS-spectra with that of the analytical standards or by comparison of mass spectra with the database integrated to the Xcalibur software.

### 2.7. GC-MS-Olfactometry 

Aroma-active compounds from cactus seed oil were characterized using an olfactometry detection port (ODP) (Sniffer 9000 system, Brechbühler, Scientific analytical solution, Schlieren, Germany) in combination with the above-mentioned PTA 3000 dynamic headspace system with GC-MS (Thermo Scientific, Darmstadt, Germany). In brief, 3.0 g of oil was transferred into a 20 mL headspace vial before dynamic headspace analysis was started as described before. For reliable results, the smell of the compounds from each sample was evaluated by three test persons. Retention time, aroma description, and intensity of all compounds causing a sensory stimulus were recorded by the tasters. Peaks that cause a sensory stimulus were assigned to the corresponding peaks in the chromatogram based on their retention time. Only volatile compounds detected by at least two test persons were identified as aroma-active.

### 2.8. Expression of Results and Statistical Analysis

For the oils obtained from the roasting process, the results were expressed as relative ratio corresponding to the sample Sidi Ifni 0 min heating time as 100%.

The samples were analyzed in triplicate, and the results were expressed as mean ± standard deviation.

The statistical analysis of roasting results was carried out by using Tukey–Kramer HSD, *p* < 0.05 were considered as significant with 95% of confidence (JMP 14.3.0, SAS Institute Inc., Cary, NC, USA).

## 3. Results and Discussion

The interplay of the individual aroma-active substances determines the sensory perception of edible oils. These substances cause a stimulus of the olfactory epithelium that is a piece of tissue the size of a stamp, located high in the nasal cavity [30]. In most cases, the flavor impression that is perceived as a single sensation is a complex sensory impression of many individual substances in a specific concentration ratio [31]. These volatile aroma-active components are of interest because they directly affect the sensory quality of the oil by their appearance, and finally, they influence consumer attitudes [31].

### 3.1. Volatile Compounds of Cactus Seed Oil

Gas chromatography−mass spectrometry−olfactometry (GC−MS−O) was used for the analysis and characterization of volatile compounds and key odorants of Moroccan cactus seed oil (*Opuntia ficus-indica*) from different locations. To the best of our knowledge, no research has been published to establish the volatile substances of cactus seed oil by GC−MS and to detect the aroma-active compounds by olfactometry. 

In the present study, a total number of 32 compounds has been detected of which 18 compounds were found to be detectable in at least 50 percent of all samples. These compounds belong to seven classes of compounds: hydrocarbons, aldehydes, alcohols, ketones, acids, esters, and isocyanides. From these volatile compounds 11 substances were identified as aroma active compounds by olfactometry (Table 1).

**Figure 2:**
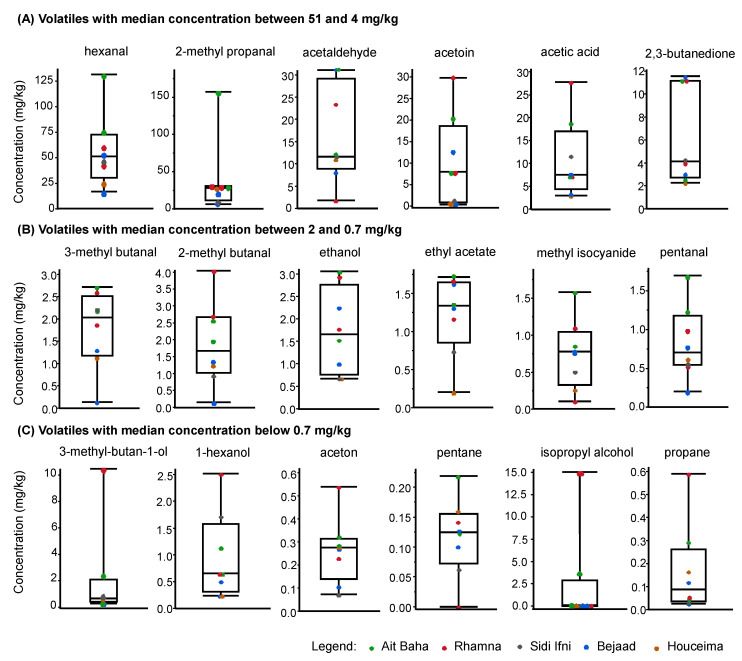
Box plot diagrams of volatile compounds extracted from cactus seed oil from different locations in Morocco by dynamic headspace GC and detection by FID. The volatiles are sorted by their median concentration, starting with the largest concentration. (**A**) shows the volatiles with the highest median concentration ranging from 51 to 4 mg/kg; (**B**) volatiles with a median concentration between 2 and 0.7 mg/kg and (**C**) volatiles with median concentration below 0.7 mg/kg).

The main volatile compounds identified in cactus seed oil were hexanal and 2-methyl propanal with average amounts of 57.4 mg/kg and 38.9 mg/kg, respectively (Figure 2). In addition, acetaldehyde (16.2 mg/kg), acetic acid (10.9 mg/kg), acetoin (10.2 mg/kg) and 2,3-butanedione (6.3 mg/kg) were found in average amounts higher than 5 mg/kg. All these compounds were identified as aroma active and may contribute to the smell of cactus seed oil. 

Hexanal is an important reaction product resulting from the oxidation of oleic, linoleic, and linolenic acid and might be formed during the processing and cleaning of the oil, when the oil is coming into contact with air. For virgin rapeseed oil Matheis and Granvogl [18] also described hexanal as one of the major volatile compounds extracted from the oil. The aldehyde 2-methyl propanal is known as volatile compound formed either by the metabolism of microorganisms following the Ehrlich pathway [32] or by Strecker degradation of α-amino acids [33]. The same formation pathway is known for 2-methyl butanal and 3-methyl butanal that were found in the oil in average amounts of 1.9 mg/kg and 1.8 mg/kg, respectively. Both compounds are also known as metabolites of Stapylococcus, Bacillus, and Paenibacillus species [16,34] and are formed as intermediates during the formation of 2- and 3-methyl-1-butan-1-ol from amino acids [16,34]. These compounds were also found in virgin rapeseed oil especially when the raw material has been stored under moist conditions. With increasing storage time, the amount of the aldehydes in the resulting oil increased significantly [20]. Acetaldehyde is produced by partial oxidation of ethanol resulting from the metabolization of carbohydrates by yeasts. Acetoin (3-hydroxy-2-butanone) is also described in the profile of volatile compounds obtained from virgin rapeseed oil [14]. The compound can be formed as an intermediate of the metabolism of some bacteria within the anaerobe degradation of glucose [35]. 

Figure 2 shows that the location of cultivation of cactus seeds strongly influences the pattern of the volatile compounds of the corresponding cactus seed oil. All identified compounds showed a huge variation. The boxplots showed that especially cactus seed oil extracted from seeds obtained from Rhamna (total amount of selected compounds: average: 165.6 mg/kg) and Ait baha (total amount of selected compounds: average: 269.4 mg/kg) contained the highest amounts of volatile compounds while oil from seeds from Bejaad (total amount of selected compounds: average: 93.3 mg/kg) only contain lower amounts. The conditions at the locations cannot explain these differences since Bejaad and Rhamna are relatively close together more in the North of Morocco while Ait Baha is located more in the South (Figure 1). Probably other influences affect the content of volatile compounds in cactus seed oil from different locations. 

In the literature, different publications on the composition of volatile compounds from fruits of *O. ficus-indica* are available. For fruits of Italian origin, Arena et al. [24] identified sixteen volatile compounds by GC-MS with E-2-hexen-1-ol and hexan-1-ol as main representatives, which amounted to about 80% of the total weight of the extracts obtained by SPME. Other compounds extracted by SMPE were (E)-2-hexenal, (Z)-2-penten-1ol, (Z)-3-hexen-1-ol, (E)-2-hexen-1-ol, (E)-2-nonen-1-ol, and (E,Z)-2,6-nonadien-1-ol. In addition, they found in extracts obtained by liquid–liquid extraction 2-methylbutanoic acid methyl ester, hexanal, 2-hydroxybutanone, linalool, (Z)-3-nonen-1-ol, (Z,Z)-3,6-nonadien-1-ol, salicylic acid methyl ester, hexanoic acid, and octanoic acid. These volatile compounds found in the fruits are completely different from the result of the present investigation. Only hexanal and 1-hexanol (1.0 mg/kg) were also identified in cactus seed oil. Reasons could be the different extraction techniques for the volatile compounds but also a change of the volatile compounds from the fruits to the oil. Agozzino et al. [25] investigated the aroma compounds present in the headspace of homogenized slurries of fresh fruits from *O. ficus-indica* from Sicilian cultivars by SPME combined with GC-MS. They also found 2-hexen-1-ol, 2-nonen-1-ol and 1-hexanol as most abundant compounds extracted from *O. ficus-indica* fruits. These compounds are known to have a fruity smell. Similar results were described by Andreu-Coll et al. [27] who isolated 35 compounds from the fruits pulp of six prickly pear cultivars with nonanol, 2,6-nonadienal, 1-hexanol, 2-hexenal, and D-limonene as predominant compounds. Aldehydes, alcohols, and terpenes were the predominant chemical families. Oumato et al. [36] identified as volatile compounds of whole cactus fruit forty-six compounds with 2-hexanal and n-hexanol, 3-methylbutanal, and 2-methylpropanal as most abundant compounds. 3-methylbutanal and 2-methylpropanal as degradation products of the metabolism of microorganisms were also found in the oil in the present study. In contrast, Rodriguez et al. [26] investigated the volatile compounds in different Opuntia fruit pulp, and they found undecane, 2-phenylethyl alcohol and decane as main components in *O. ficus-indica* while the amount of 2-hexen-1-ol or 1-hexanol was not detectable or low. In another publication on the volatile compounds extracted from four developmental stages of *O. ficus-indica* flowers, Ammar et al. [37] identified as main compounds carboxylic acid (28–97%), terpenes (0.2–57%), esters (0.2–27%), and alcohols (<1.8%).

In addition, Table 1 shows that the number of volatile compounds identified in cactus seed oil was much higher than that in the press cake or the seeds. For the seeds, only acetaldehyde, 2-methyl propanal, 2-methyl butanal, and hexanal were detected while from the press cake acetaldehyde, acetic acid, pentanal, and propane were extracted. The higher number of volatile compounds in the oil may be explained by the fact that the oil and the volatile compounds in seeds or press cake are partly still protected in the cells, and the volatile compounds are therefore not extracted.

### 3.2. Investigation of Volatile Compounds of Cactus Seed Oil Formed during Roasting of Cactus Seeds

Roasting is the action of exposing food to direct or indirect heat forming, in a non-enzymatic reaction, pigments with specific yellow-brown color and aroma active compounds [38]. One reason for this procedure is to produce pleasant aroma components resulting in a roasty and nutty perception of the food accepted by consumers [39]. The application of seed roasting has been known for a number of oil seeds, such as sesame [40], pine nut [41], pumpkin [42], or rapeseed [43]. Depending on the temperature and the time, this heat treatment results in the formation of Maillard reaction products or degradation products of oxidation that are responsible for the typical taste and smell of edible oils from roasted oil seeds. In the Maillard reaction reducing sugars and amino acids react at temperature of about 120 °C, and the absence of water to numerous aroma active reaction products. Siegmund and Murkovic [39] showed that compounds that are responsible for roasty/nutty aroma notes require a roasting temperature of at least 90 °C. At an even higher temperature of about 170 °C sugars break down and form compounds with a characteristic color and flavor. This process is known as caramelization. Since oil is a very good carrier for short-chain aroma active compounds, many of the formed compounds are co-extracted with the oil during oil processing.

In the present study, the effect of roasting cactus seeds at 110 °C for different times on the profile of the volatile compounds of the resulting oils was investigated. The volatile compounds that appear during the roasting process and theodor impression named by the test persons are reported in Table 2.

The profile of the volatile compounds obtained from oil of roasted cactus seeds changed remarkably in comparison to the oil from unroasted seeds. While oil from unroasted cactus seeds was dominated by aldehydes, alcohols, and ketones in oil from roasted seeds, a lot of Maillard reaction products such as different pyrazines, dimethyl-trisulfide, or 5-methyl furfural have been detected.

**Figure 3:**
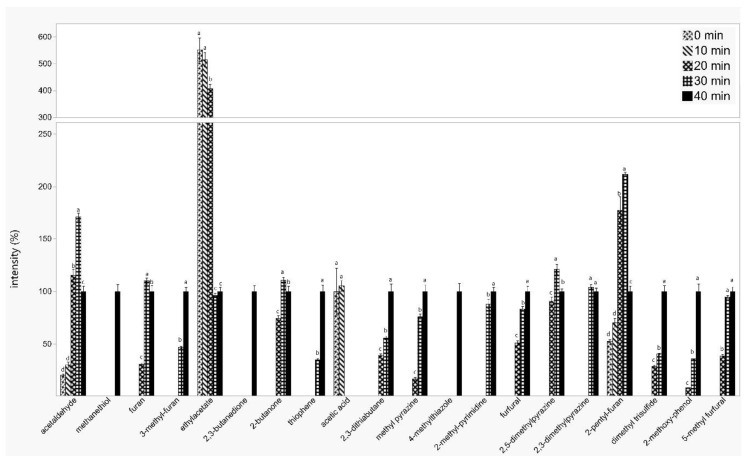
Volatile compounds of cactus seed oil after roasting of the seed material and extraction of the oil. Shown are the relative amounts in comparison to 40 min of roasting (100%), except for acetic acid (100% ≙ 0 min) (Mean ± SD, *n* = 3). Values with different letters are significantly different in comparison to the other time points by applying Tukey–Kramer HSD test (*p* < 0.05).

Comparing the aroma profile of oils produced from non-roasted and roasted seeds revealed the presence of twenty volatile compounds that appear or disappear during the roasting process at different times (Table 2). The percentage of the compounds changed depending on the roasting time are presented in Figure 3. Twelve of those compounds were identified as aroma active compounds by olfactometry analysis (Table 2).

Figure 3 shows that most of the volatile compounds increased during roasting with increasing roasting time such as 3-methyl furan, acetic acid, 2,3-dithiabutane, methyl pyrazine, 4-methylthiazhole, 2-methyl pyrimidine, furfural, dimethyl trisulfide, 2-methoxy phenol, and 5-methyl furfural. On the other hand, the amount of ethyl acetate is continuously reduced with roasting time, and after 40 min of roasting, only about a quarter was detectable. Some of the compounds are only formed after 40 min of roasting such as methanethiol, 2,3-butanedione, or 4-methylthiazole. Acetaldehyde, furan, 2-butanone, 2,5-dimethyl pyrazine, 2,3-dimethyl pyrazine, and 2-pentyl furan showed an increase until 30 min of roasting, and at a higher temperature, the concentration decreased.

The formation of 2, 3-dimethyl pyrazine, 2, 5- dimethyl pyrazine, thiophene, 3-methyl furan, furan, 5-methyl furfural, 2-pentyl furan, methyl pyrazine, 2-methyl pyrimidine, 2-methoxy phenol, and furfural during the roasting process can be explained by the Maillard reaction. Lee et al. [44] showed that different Maillard reaction products are formed during roasting of perilla seeds depending on the roasting conditions with 2-methylpyrazine and 2,5-dimethylpyrazine as main products. In rapeseed oil, the amount of pyrazines (110-fold) and aldehydes (65%) increased drastically after roasting of the seeds in comparison to the initial content [43]. Gracka et al. [21] identified in oil from roasted rapeseed 2-methyl butanal, 1-methyl-1H-pyrrole, 2,5-dimethyl pyrazine, and methyl pyrazine as important compounds with 2-methyl butanal as one of the common Strecker degradation products, and 2,5-dimethyl pyrazine and methyl pyrazine as Maillard reaction products. El Monfalouti et al. [45] also showed an increase of Maillard reaction products such as pyrazines of furans with increasing roasting time.

Methanethiol, thiophene, dimethyl trisulfide, 2, 3-dithiabutane and 4-methyl thiazole are sulfur compounds that are also formed during the Maillard reaction. In addition to the Maillard reaction, which involves a thermal degradation, methionine or cysteine may degrade by Strecker degradation pathway to form dimethyl sulfide, another immensely powerful flavor compound. Methanethiol may also further react forming mono-, di-, and trisulfides. For ethyl acetate, the decrease during the roasting process might be explained by degradation or evaporation processes due to the temperature.

The olfactometric analysis of the oils obtained at different times from the roasting process was in accordance with the analytical results. The intensity of the aroma increased during roasting for the following compounds: furan, furfural, 4-methyl thiazol, and 2-methoxy phenol (Table 3).

## 4. Conclusions

During the last decade, growing interest in the use of different plant parts of *O. ficus-indica* arose, resulting in a large number of scientific papers on the composition of the flowers, fruits, and seeds [5,6,9,10,11,12,13,14,15,16,17,18,19,20,21,22,23,24,25]. To the best of our knowledge, all these studies dealt with seeds and fruits of prickly pear, but only few studies took cactus seed oil into account [6,9,10].

In the present study, cactus seed oil from five regions of Morocco and from different times of seed roasting at 110 °C was used to investigate for the first time the profile of volatile compounds of cactus seed oil and to show the increase and decrease of volatile compounds during roasting. The smell of cold-pressed cactus seed oil from sound seeds is mainly influenced by the occurrence of aldehydes, and during roasting, Maillard reaction products, especially pyrazines and sulfur-containing compounds, dominate the profile of cactus seed oil. The intensity of the smell of cactus seed oil from roasted seeds can be influenced by the roasting time. With increasing roasting time, the amount of the volatile and aroma active compounds increases for most of the compounds resulting in a higher intensity of the smell. From the results obtained, we concluded that the origin of the oil has a qualitative and quantitative influence on volatile compounds. Our results also show that the roasting time of cactus seeds promotes the formation of volatile organic compounds. These compounds are responsible for the aroma of the final product.

This investigation might be a first step in revealing the profile of volatile compounds from cactus seed oil obtained from untreated and roasted seeds, respectively. This should be useful for supporting the sensory assessment of cactus seed oil in the future, but more information about the influences of cultivation conditions, varieties, or processing conditions is necessary. It is also necessary to know more about the aroma profile of the oil, describing the influence of the different aroma active compounds more in detail. In addition, it would be useful to get more information about the contribution of the individual aroma compounds to the overall aroma of cactus seed oil by GC-olfactometry with aroma extract dilution analysis. This would allow to identify aroma compounds that are responsible and typical for virgin cactus seed oil. In this connection, the interaction of the different volatile compounds would also be interesting in order to understand how the typical aroma is created. Finally, the increasing knowledge on the composition of volatile compounds of cactus seed oil and the importance of certain volatile compounds for the flavor of the oil can be used to develop analytical methods for the identification of the authenticity of virgin cactus seed oil which, due to its high price, is particularly at risk of being adulterated with cheaper oils.

## Figures and Tables

**Table 1:** Occurrence of volatile compounds of cactus seed oil, cactus seeds, and cactus press cake.

Volatile Compound	Cactus Oil	Cactus Seeds	Cactus Press Cake	Odor Impression
**Aldehydes**				
acetaldehyde	+	+	+	ether, old cheese
2-methyl propanal	+	+		chocolate
3-methyl butanal	+			cheese, moldy
2-methyl butanal	+	+		chocolate, cocoa
pentanal	+		+	green
hexanal	+	+		grass, green
**Alcohols**				
ethanol	+			alcohol
isopropyl alcohol	+			solvent
isopentyl alcohol	+			-
**Ketones**				
acetone	+			-
2,3-butanedione	+			fruit, sweet
acetoin	+			-
**Acids**				
acetic acid	+		+	vinegar
**Esters**				
ethyl acetate	+			-
**Hydrocarbons**				
pentane	+			-
hexane	+			-
propane	+		+	-
**Isocyanides**				
methyl isocyanide	+			-

**Table 2:** Volatile compounds specific to roasting of cactus seed oil.

Volatile Compound	Odor Impression
acetaldehyde	ether, old cheese
methanthiol	-
furan	coffee
3-methyl furan	coffee
ethylacetate *	-
2,3- butanedione	fruit, sweet
2- butanone	fruit, sweet
thiophene	-
acetic acid	vinegar
2,3- dithiabutane	-
4-methyl thiazole	green
methyl pyrazine	-
2-methyl pyrimidine	-
furfural	baked potatoes
2,5- dimethyl pyrazine	-
2,3- dimethyl pyrazine	-
2-pentyl furan	-
dimethyl-trisulfide	onion
5-methyl furfural	almond
2-methoxy phenol	smoke

* aroma was perceived by the testers but no specific odor impression was given.

**Table 3:** Aroma intensity of some aroma volatile compounds.

	0 min	10 min	20 min	30 min	40 min
furan	−	−	−	+	++
furfural	−	−	++	+++	+++
4-methyl thiazol	−	−	−	−	++
2-methoxy phenol	−	−	−	−	+++

No detection (−), low intensity (+), medium intensity (++), high intensity (+++).
